# Left ventricular diastolic dysfunction of the cardiac surgery patient; a point of view for the cardiac surgeon and cardio-anesthesiologist

**DOI:** 10.1186/1749-8090-4-67

**Published:** 2009-11-24

**Authors:** Efstratios E Apostolakis, Nikolaos G Baikoussis, Haralabos Parissis, Stavros N Siminelakis, Georgios S Papadopoulos

**Affiliations:** 1 Cardiothoracic Surgery Department, University of Patras, School of Medicine, Patras, Greece; 2 Cardiac Surgery Department, University of Ioannina, School of Medicine, Ioannina, Greece; 3 Basildon & Thurrock University Hospital NHS FT, Basildon, Essex, UK; 4 Department of Clinical Anesthesiology and Intensive Postoperative Care Unit, University of Ioannina, School of Medicine, Ioannina, Greece

## Abstract

**Background:**

Left ventricular diastolic dysfunction (DD) is defined as the inability of the ventricle to fill to a normal end-diastolic volume, both during exercise as well as at rest, while left atrial pressure does not exceed 12 mm Hg. We examined the concept of left ventricular diastolic dysfunction in a cardiac surgery setting.

**Materials and methods:**

Literature review was carried out in order to identify the overall experience of an important and highly underestimated issue: the unexpected adverse outcome due to ventricular stiffness, following cardiac surgery.

**Results:**

Although diverse group of patients for cardiac surgery could potentially affected from diastolic dysfunction, there are only few studies looking in to the impact of DD on the postoperative outcome; Trans-thoracic echo-cardiography (TTE) is the main stay for the diagnosis of DD. Intraoperative trans-oesophageal (TOE) adds to the management. Subgroups of DD can be defined with prognostic significance.

**Conclusion:**

DD with elevated left ventricular end-diastolic pressure can predispose to increased perioperative mortality and morbidity. Furthermore, DD is often associated with systolic dysfunction, left ventricular hypertrophy or indeed pulmonary hypertension. When the diagnosis of DD is made, peri-operative attention to this group of patients becomes mandatory.

## Introduction

Left ventricular diastolic dysfunction (DD) is defined as the inability of the ventricle to fill to a normal end-diastolic volume, both during exercise as well as at rest, while left atrial pressure does not exceed 12 mm Hg [1-3]. It has been shown that several patients with DD are suffering from paroxysmal dyspnoea and "unexplained" pulmonary oedema with a normal ejection fraction [4,5]). Among patients operated for coronary artery disease or aortic stenosis, the incidence of left ventricular DD ranges widely between 44%, and 75% [6-10]. The significance and the severity of ventricular diastolic dysfunction among these patients are not well elucidated. On the other hand, estimation of the degree of DD peri-operatively, is difficult in up to 20% of cardiac-surgery patients for several reasons [10,11] including rhythm abnormality, preload and afterload alterations, coexistence of valvular disease, age related changes, and inability to obtain proper Doppler images [12-15]. The diastolic heart failure annual mortality varies between 9-28% (four-fold that of disease-free subjects [16], while it has also been linked to increased incidence of postoperative complications (mortality or morbidity) after cardiac surgery [13,17,18]. Revascularization of ischemic myocardium seems to be beneficial for DD (if not immediately), some weeks after revascularization [19]. Potential direct postoperative improvement in diastolic function may be offset by the detrimental effect of global ischemia during cardioplegic arrest in combination with myocardial interstitial oedema [11,20]. There are only a few studies concerning surgical outcomes of patients suffering from diastolic dysfunction. Moreover, intra-operative diagnosis and strategies to manage patients with left ventricular diastolic dysfunction are not well clarified. In that sense, diastolic dysfunction could be considered perioperatively as a "Trojan horse".

### Source of Research

Pertinent medical literature in the English language was identified through a Medline computerized literature search and a manual search of selected articles using the key words "left ventricular diastolic dysfunction", "left ventricular diastolic impairment", "transmitral flow Doppler", "pulmonary venous flow patterns". The search terms were combined using the Boolean operator term "or" to find all abstracts pertaining to the chosen search terms. These individual terms were then combined using the Boolean operator term "and" to find articles that contained information of all search terms. The reference lists of articles found through these searches were also reviewed for relevant articles. Links provided on the web sites of published articles were searched for relevant articles.

### Pathophysiology

**Figure 1:**
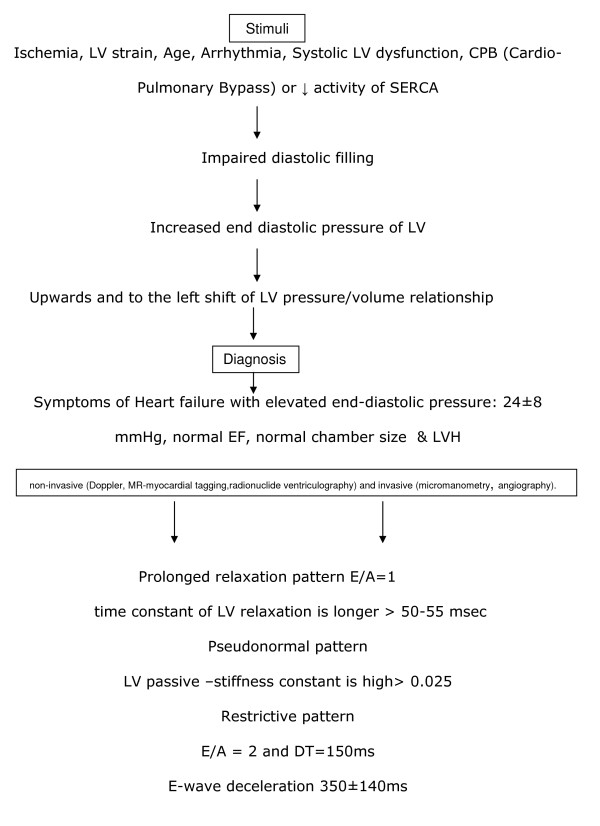
**Pathophysiology of DD and its consequences**.

DD is present when an elevated filling pressure is necessary to achieve normal ventricular filling. So, DD is related to abnormal left ventricular relaxation and filling during diastolic phase of cardiac cycle [21-24]. During this phase there are four timely and sequential events: a) isovolemic relaxation, b) rapid (early) LV filling, c) slow LV filling (diastasis) and d) atrial contraction [2,23]. In figure 1 is shown schematically the pathophysiology of DD. According to echocardiographic depiction, filling of normally relaxed LV is completed in two phases: the first phase is due to the passive filling of the LV, is massive and depicted early in diastole by a high E wave. The second phase is due to the left atrial contraction, takes place during late diastolic phase, and leads to late LV filling depicted by the wave A of transmitral inflow Doppler [22,25]. The rate of decrease of E wave in early diastole depends on the rate of increase in LV pressure and is represented by the so-called deceleration time (DT). This time is influenced by a number of factors such as, **a) **left atrial-left ventricular pressure gradient at the time of mitral valve opening, **b) **left atrial chamber compliance, **c) **left ventricular chamber compliance, **d) **grade of left ventricle relaxation, **e) **visco-elastic forces of the myocardial wall, **f) **pericardial restraint and finally **g) **left-right ventricular interaction. Left ventricular relaxation-similar to contraction- is an energy-dependent process, because it requires the re-uptake of calcium into the sarcoplasmic reticulum [26]. When patients with left ventricular DI are subjected to stress-as occurs during surgery or during faster heart rates-due to shorter diastolic filling time available, the ventricle is not allowed to relax and fill properly; thus, causing increased left ventricular end-diastolic pressure and pulmonary congestion [1,2,16]. Furthermore, relaxation of the left ventricle is determined by visco-elasticity and restoring forces (recoil). It is believed that impaired diastolic filling of the left ventricle is the first manifestation of active ischemia and results in an upward shift of left ventricular diastolic pressure-volume relationship [2,26]. Decreased activity of sarcoplasmic reticulum calcium ATPase pump (SERCA) can slow down calcium removal out of the cytosolic net [27]. In contrast, increased levels or activity of phospholamban-the natural SERCA-inhibitory protein-can also impair relaxation. Hypothyroidism decreases SERCA and increases phospholamban, leading to impaired relaxation, while the opposite effect occurs in hyperthyroidism [27]. In a similar way, increasing the action of SERCA by administration of captopril, and β-agonists (or decreasing the action of phospholamban), results in improvement of diastolic relaxation [28].

### Pathophysiology and diagnosis of DD

Another aspect of DD is the relationship between systolic and diastolic left ventricular dysfunction [2,29,30] Increased left ventricular end systolic volume for example, affects the rate of left ventricular relaxation, and as a result, patients with reduced LV ejection fractions are expected to have a prolonged relaxation time [2]. Loading conditions, such as inotropic stimulation and neurohumoral factors generally affect both systolic and diastolic function in a parallel way [2]. As it has been shown, elevated left ventricular end-diastolic pressure may or may not be associated with systolic dysfunction of left ventricle, suggesting left ventricular DD even in the absence of reduced left ventricular ejection fraction [29]. Indeed, patients with symptoms of heart failure and normal ejection fraction have significant abnormalities in active relaxation and passive stiffness, which cause increased left ventricular end-diastolic pressure [30]. Literature review: "The theory" of DD is presented in table 1.

**Table 1:** Articles "investigating the background" of the entity Diastolic Heart Failure (DHF).

Author	Year	Journal	Conclusions
Kessler KM et al [35]	1989	Hosp Pract	Introduction of the term DHF

Paulus WJ [67]	1999	Cardiovasc Res.	Development of specific diagnostic criteria for DHF

Bruch C et al [68]	2000	Eur Heart J	Tei-index: relation to LVEDP, sensitive indicator of overall cardiac dysfunction

Mandinov L et al [69]	2000	Cardiovasc Res	Doppler--Echo definitions

Vasan and Levy [70]	2000	Circulation	Development of criteria for definite, probable and possible DHF

Crossman W [16]	2000	Circulation	Thoughtful Editorial

Zile MR et al [71]	2001	Circulation	Tested the hypothesis that measurements of the LV relaxation and passive stiffness were not necessary to make the diagnosis of DHF

Poulsen SH et al [72]	2001	Dan Med Bull	DHF following acute MI

Catuzzo B et al [73]	2003	J Card Fail	Regarding patients with CHF: BNP plasma levels is related to diastolic restrictive pattern

Hogg K et al [21]	2004	J Am Coll Cardiol	Epidemiology of the syndrome of heart failure with preserved LV systolic function: clinical characteristics

Zile MR et al [30]	2004	N Engl J Med	Invasive assessment of DHF. Identification of significant abnormalities in active relaxation and passive stiffness

Yturralde RF et al [74]	2005	Prog Cardiovasc Dis	Review and current recommendations

Zile MR et al [75])	2005	Prog Cardiovasc Dis	Overview of systolic and DHF

Shammas RL et al [76]	2007	Int J Cardiol	DHF: "what we don t know"

Scardovi AB et al [77]	2007	Eur J Echocardiogr	BNP and advanced DHF

Literature review: "The Theory" of DD

### Diagnosis of DD in a Cardiothoracic setting

Assessment and diagnosis of DD can be performed with non-invasive (2D and Doppler-echocardiography, colour Doppler M-mode, Doppler tissue imaging, MR-myocardial tagging, radionuclide ventriculography) and invasive techniques (micromanometry, angiography, conductance method). Typical findings of primary DD in chest radiograph include absence of cardiomegaly and presence of pulmonary congestion. Electrocardiogram/ECHO reveals the presence of excessive concentric hypertrophy in combination with normal ejection fraction [5]. Measurement of peak early filling wave (E wave: is caused by difference in atrium-ventricle pressure) and atrial filling wave (A wave: is caused by atrial contraction) ratio by Doppler echocardiography, as well as deceleration time (DT: is caused by left ventricular compliance), are useful screening tools for abnormal left ventricular relaxation [29]. In presence of abnormal relaxation, atrial contraction occurs in an incompletely empty atrium and blood is propelled into the left ventricle in increased velocity, accounting for the heightened A wave and consequent decreased E/A ratio. Blood flow in the pulmonary veins is biphasic, with peaks of forward flow occurring in both systole and diastole and inverse diastolic flow occurring during atrial contraction. There is an inverse relationship between left atrial pressure and pulmonary venous systolic flow. That is the reason why determination of systolic pulmonary venous flow velocity is a rapid method to estimate LV filling pressures after CABG [30].

### Pathological filling is determined from transmitral flow pattern

1) Prolonged relaxation pattern: characterized by prolonged isovolumetric relaxation time and deceleration time, low E and high A wave velocities with an E/A wave ratio typically 1. It is related to the remodelling process including hypertrophy or scarring of an infarct zone leading to a non-uniform LV relaxation.

2) Pseudonormal pattern: an intermediate stage between prolonged relaxation and restrictive filling as a consequence of disease progression. There is an association with atrial dilatation and prominent pulmonary venous wave reversal.

3) Restrictive pattern: associated with shortened isovolumetric relaxation time, increased peak E wave velocity with very short deceleration time and small A wave, leading to an E/A wave ratio of 2. This pattern might be due to increasing LV volume and also to increased myocardial stiffness. DD is severe when the transmitral filling pattern E/A ratio is 2 and the deceleration time is 150 ms.

For patients undergoing cardiac surgery, Doppler assessment of transmitral flow has been used to estimate postoperative left ventricular filling pressure, relaxation, and stiffness [31]. The most important problem in evaluating transmitral flow patterns is their great variation, depending on many factors such as: heart rate [32,33], preload [34], afterload [34], positive-pressure mechanical ventilation [21], systolic ventricular function [35,36], use of inotropic or generally vasoactive agents due to their effect on the afterload [34,37], and hemodilution (higher velocities due to reduced blood viscosity) [34]. To surmount this, a new method for diagnosis of LV diastolic dysfunction, the so called flow propagation velocity (Vp) is applied. It bears the advantage of being insensitive to heart rate and preload changes [10]. According to Vp measurement, left ventricular filling patterns does not change significantly after cardiopulmonary bypass. Furthermore, newer techniques such as tissue Doppler imaging (TDI) which measures high intensity, low velocity echo of the myocardium has been developed. By using TDI, local myocardial relaxation can be calculated by obtaining the velocity of early diastolic wall motion (Em) and it's timing [38]. In other words, TDI allows assessment of diastolic function because of its unique ability to assess regional abnormalities in relaxation, in addition to their global effect on ventricular relaxation and filling dynamics. An E/Em ratio > 10 remains the best discriminatory value when it is used as a single parameter for the prediction of elevated filling pressures or simply diastolic dysfunction [39]. However, definite diagnosis of diastolic dysfunction is established by cardiac catheterization and direct measurement of pressure at the end of systole and volume loops [40]. This invasive assessment of diastolic function allows the study of isovolumic relaxation (time constant of LV relaxation is longer > 50-55 msec) and evaluation of the passive elastic properties of the myocardium (LV passive-stiffness constant is high).

### Intraoperative diagnosis

Intraoperative diagnosis of diastolic dysfunction is difficult, [41,42] because: **a) **most variables measuring diastolic function depend on loading conditions, heart rate and age [32-34,43], **b) **no single individual measurement can fully characterize left ventricular diastolic dysfunction, and **c) **ECHO estimation may give different results whether it is performed with the patient awake and breathing spontaneously, or anesthetized and receiving positive pressure ventilation [35]. Diastolic dysfunction of left ventricle can be intraoperatively diagnosed, estimated and graded by using Trans Oesophageal Echo (TOE). Moreover, valuable information may be obtained with the additional use of a Swan-Ganz catheter [33,34,39]. According to Ranucci [44], first degree of diastolic dysfunction of the left ventricle is depicted as impaired relaxation, is usually observed just after discontinuation of cardiopulmonary bypass, and is often reversible (temporary). Second degree mimicking pseudo-normalization, is a more severe condition, which sometimes is an intermediate step towards, third degree of dysfunction which is characterized by a restrictive pattern. An increased ratio (> 2) between E and A waves of transmitral flow, and a blunted systolic waveform of the pulmonary vein flow is present due to left atrial pressure [34,36,39]. It has been demonstrated that mitral and pulmonary vein flow indexes correlate with pulmonary capillary wedge pressure (PCWP) [44,45]. Therefore, additional measurement of PCWP by using a Swan-Ganz catheter may be in this phase useful in estimating the time course of diastolic dysfunction and the effect of therapeutic manipulations [44]. Fluid responsiveness is better defined by TOE derived variables (left ventricular end-diastolic area, peak blood velocity variation), but some information can be derived by the Swan-Ganz catheter as well (PCWP and peak pulmonary pressure variation) [45,46]. In table 2 we present the high risk groups for developing DD, while in table 3, we report articles looking into: the impact of diastolic dysfunction (DD) on patient's outcome following Cardiac Surgery.

**Table 2:** High risk groups for developing DD

Systolic dysfunction	Only 50% to 60% of patients with clinical findings of congestive heart failure have an abnormal systolic function, which is indicated by reduced ejection fraction. The remaining 40%-50% of pts, have congestive heart failure with normal systolic function and represent the patients with diastolic dysfunction [22,23]. For clarification, Sanderson proposed the term "heart failure with normal ejection fraction" (HFNEF) for left ventricular diastolic dysfunction, and heart failure with reduced ejection fraction (HFREF) for systolic dysfunction of left ventricle [78]. According to this classification, the main difference between HFNEF and HFREF is the degree of ventricular remodeling accompanied by increased ventricular volume in HFREF [78]. In other words, distinction between systolic and diastolic dysfunction is very important because the latter has a lower mortality (5%-8% annually), and requires different medical management (no inotropes) [22,23].
LVH	In patients with AS, preoperative DD is attributable to hypertension, myocardial hypertrophy- fibrosis, and/or to ischemia [64].

CAD	Patients with CAD are prone for the development of postoperative myocardial diastolic dysfunction [39]. Left ventricular filling abnormalities have been detected in as many as 90% of patients [39]. Possible related factors that were considered were ischemia, hypertrophy, and hypertension [79].

DM	All insulin--dependent diabetes mellitus patients with left diastolic dysfunction had evidence of definite autonomic neuropathy [80]. Moreover, diabetic patients with autonomic neuropathy form a subgroup of particularly high mortality and cardiovascular event risk [81,82].

Age	Aging is correlated to DD through an increase upon wall thickness (secondary to enlargement of cardiac myocytes), and changes in the vasculature, the diameter, and vascular stiffness of the aorta and large arteries [83]. Up to 60% of geriatric patients with normal EF, following non-cardiac surgery, had been postoperatively diagnosed with diastolic dysfunction [35].

High risk groups for DD

**Table 3:** Articles looking into: The impact of diastolic dysfunction (DD) on patient's outcome following Cardiac Surgery.

Authors	Year	Journal	Conclusions
Casthely et al [84]	1997	J Thorac Cardiovasc Surg	The effects of myocardial protection on diastolic function after cardiac operations

Bernard F et al [13]	2001	Anesth Analg	The significance of diastolic dysfunction perioperatively; Diastolic dysfunction is associated with difficult weaning from CPB.

Vaskelyte J [18]	2001	Eur J Echocardiogr	The interesting concept to subdivide patients with severe LV dysfunction into different groups according to diastolic filling pattern abnormality. One of the few articles investigating the relationship between diastolic dysfunction and post-operative mortality. Drawbacks: All patients had low EF < 35%.

Liu J et al [17]	2003	Am J Cardiol	The prognostic value of transmitral flow patterns on patients following CABG; Probably one of the most important papers on the subject. The study claims that pseudonormal and restrictive TMF patterns, correlates with short term adverse outcome

Malouf PJ [85]	2006	J Am Soc Echocardiogr	Doppler tissue imaging of mitral annular velocity: Lateral segmental velocity has advantages over the septal segmental velocity

Literature review: the Outcome

### Progression of DD following Cardiac surgery

Following coronary artery bypass grafting, DD is temporarily deteriorated (expressed as a decrease in E-max and an increase in A-max of transmitral flow) [47]. This deterioration of DD seems to persist, at least for the first three postoperative hours after coronary artery bypass grafting [48,49]. In a similar way, Yamamoto et al by using classical ECHO after coronary artery bypass grafting, showed that DD was characterized by a decrease in E wave velocity, prolongation of the E wave DT, and a decrease of E/A ratio [43]. Potential implicated mechanisms are those of free oxygen radicals, altered intracellular calcium homeostasis, or both [50,51]. Temporary improvement has been shown, especially if calcium channels blocking factors like diltiazem were perioperatively administered or added in the cardioplegic solution [43,50,52,53]. For patients who underwent off-pump coronary artery bypass grafting (OPCAB), comparative studies on the postoperative changes in left ventricular diastolic function, have shown that, while left ventricular diastolic dysfunction impairment was observed in both groups (conventional CABG and OPCAB), it was more significantly impaired in the CABG group [54]. Other studies showed that right ventricular diastolic dysfunction was in a similar way significantly impaired after CABG and OPCAB [43,55,56], and this deterioration persisted in up to one year postoperatively [15]. In contrast to this, Shi et al who evaluated short- and long-term evolution of biventricular diastolic performance postoperatively in 49 pts who underwent coronary artery bypass grafting showed that postoperative deterioration of diastolic dysfunction had an absolute return to preoperative status at six months postoperatively [9].

### Management of DD

According to a multivariate analysis by Bernard et al [13], left ventricular diastolic dysfunction was a better predictor of hemodynamic instability after cardiac surgery compared to systolic dysfunction. Treatment of the underlying disease is currently the most important therapeutic approach. In patients with tachycardia, use of b-blockers or calcium antagonists, is beneficial so as to prolong diastolic (filling) time [24,57]. Treatment of atrial fibrillation by cardioversion or amiodarone infusion is indicated in patients with diastolic dysfunction [22,24,57]. In addition, digitalis may decelerate ventricular rate in cases of permanent atrial fibrillation, and contribute to better ventricular filling [58]. Denault et al [59] developed a diagnostic algorithm which they then applied to a group of 74 cardiac surgical patients, to determine whether moderate to severe left ventricular diastolic dysfunction (LVDD) and right ventricular diastolic dysfunction (RVDD) can predict difficult discontinuation of cardiopulmonary bypass. Patients with moderate to severe LVDD tended to have higher PCWP compared to those with normal to mild LVDD. The presence of moderate to severe RVDD was also associated with lower mean pulmonary artery pressure and lower cardiac index compared to patients with normal to mild RVDD. Difficult separation from cardiopulmonary bypass was present in 65.5% and 72% of patients with moderate/severe LVDD and RVDD respectively, in contrast to 40.9% and 48% of patients with normal/mild LVDD/RVDD. They concluded that moderate and severe degree of LVDD and RVDD can be identified with very good reproducibility, and both degrees of diastolic dysfunction are associated to difficult discontinuation from cardiopulmonary bypass [59]. During this effort, transesophageal echo is a needful tool to estimate the degree of diastolic dysfunction, as well as preload and afterload. Appropriate increase of volume load is a milestone of timing in order to discontinue cardiopulmonary bypass. Phosphodiesterase inhibitors seem to be beneficial for diastolic dysfunction improvement, and should be used in perioperatively [60]. In a similar way, Levosimendan may used in perioperative management of diastolic dysfunction [61]. It increases cardiac output and decreases pulmonary capillary wedge pressures. This mode of enhanced contractile force generation is achieved without an increase in myocardial oxygen consumption, intracellular calcium concentrations, or an adverse effect on diastolic function [61]. For the next postoperative days milestone of treatment remain diuretics, in doses which prevent dyspnea and liver congestion on one side, but not reduce the cardiac output on the other [57]. ACE inhibitors in combination with spironolactone are beneficial because they prevent excessive activation of rennin-angiotensin-aldosterone system, and improve ventricular relaxation although not yet confirmed [62,63]. In contrast to systolic dysfunction, use of calcium antagonists alone or in combination with ACE, contributes effectively in hypertension control and has a beneficial influence on hypertrophic myocardium [23,24,58]. In patients with diastolic dysfunction due to hypertrophic cardiomyopathy (either idiopathic or due to acquired aortic valve stenosis), the main problem is to load the left ventricle with adequate volume (preload) because it is common to notice an echo-finding of low preload (i.e. very low left ventricular end-diastolic area), while the measured PCWP is found high [55]. Such patients need increased volumes, but each fluid administration should be carefully guided by constant measurement of PCWP, in order to avoid an abrupt increase in pulmonary venous pressure and consequent acute pulmonary oedema [55]. Postoperatively, use of intra-aortic balloon pump in patients with left ventricular diastolic dysfunction seems to result in a favourable influence on left ventricular function [34]. Possible explanations for this effect lie on the positive effects of balloon on coronary flow against ischemia, the favourable effect on systolic function of left ventricle, and the increase of left ventricular long axis [34]. For those cases whereby "restricted pattern" is diagnosed, inotropic agents should be considered. Maslow et al showed that the use of inotropes in 44 patients, who underwent AVR for stenosis, was associated with significantly larger increase in right ventricular ejection fraction and cardiac output after CPB [64]. Changes in cardiac output and index were more strongly correlated with changes in RVEF than LVEF. Lastly, infusion of a new B-natriuretic peptide (BNP) nesiritide was associated with increased CO in patients with diastolic dysfunction and low CO syndromes undergoing cardiac surgery, when other measures failed. This agent seems to offer an additional option to inotropes and fluid challenges perioperatively [65]. Castellá et al in an experimental study conducted in pigs in 2006, demonstrated that temporary LAD ischemia alters the normal sequential pattern of contraction responsible for ejection and suction through reduction of the systolic contractile force, and prolongation of the endocardial contraction into early diastole to disrupt the normal endocardial-epicardial sequence responsible for ventricular suction [66]. The systolic and diastolic effects of myocardial stunning were studied to evaluate the role of the endocardial and epicardial segments and to determine if preconditioning by Na+-H+ exchange (NHE) inhibition effect post-stunning dysfunction. In this study conducted in Yorkshire-Duroc pigs, NHE inhibition before ischemia limits postischemic systolic and diastolic dysfunction by re-establishing the expected shortening sequences within the ventricular myocardial band model [66].

## Conclusion

There are only few studies looking in to the impact of DD on the outcome following cardiac surgery. Without doubt DD with elevated left ventricular end-diastolic pressure can predispose to increased perioperative mortality and morbidity. Furthermore, DD is often associated with systolic dysfunction, left ventricular hypertrophy or indeed pulmonary hypertension. The mainstay of management of DD starts with the prompt recognition and diagnosis of this entity and relies on the aggressive management of the underlie aetiology of this insidious disease.

## Competing interests

The authors declare that they have no competing interests.

## Authors' contributions

All authors: 1. have made substantial contributions to conception and design, or acquisition of data, or analysis and interpretation of data; 2. have been involved in drafting the manuscript or revisiting it critically for important intellectual content; 3. have given final approval of the version to be published.
